# The prevalence of schistosomiasis in Uganda: A nationally representative population estimate to inform control programs and water and sanitation interventions

**DOI:** 10.1371/journal.pntd.0007617

**Published:** 2019-08-14

**Authors:** Natalie G. Exum, Simon P. S. Kibira, Ronald Ssenyonga, Julien Nobili, Alexandra K. Shannon, John C. Ssempebwa, Edridah M. Tukahebwa, Scott Radloff, Kellogg J. Schwab, Fredrick E. Makumbi

**Affiliations:** 1 Department of Environmental Health and Engineering, Bloomberg School of Public Health, Johns Hopkins University, Baltimore, MD, United States of America; 2 Department of Community Health and Behavioral Sciences, School of Public Health, Makerere University, Kampala, Uganda; 3 Department of Epidemiology and Biostatistics, School of Public Health, Makerere University, Kampala, Uganda; 4 Department of Population, Family and Reproductive Health, Johns Hopkins Bloomberg School of Public Health, Johns Hopkins University, Baltimore, MD, United States of America; 5 Department of Disease Control and Environmental Health, School of Public Health, College of Health Sciences, Makerere University, Kampala, Uganda; 6 Vector Control Division, Ministry of Health, Kampala, Uganda; University of Washington, UNITED STATES

## Abstract

**Background:**

To improve schistosomiasis control programs in Uganda, where intestinal schistosomiasis is a widespread public health problem, a country-wide assessment of the disease prevalence among all age ranges is needed. Few studies have aimed to quantify the relationships between disease prevalence and water and sanitation characteristics across Uganda to understand the potential to interrupt disease transmission with an integrated package of interventions.

**Methodology/Principal findings:**

A nationally representative survey was undertaken that included a household and individual questionnaire followed by disease testing based on detection of worm antigens (circulating cathodic antigen–CCA), diagnosis and treatment. A comprehensive set of questions was asked of randomly sampled individuals, two years of age and above, to understand their water and sanitation infrastructure, open defecation behaviors, exposure to surface water bodies, and knowledge of schistosomiasis. From a set of 170 randomly sampled, geographically diverse enumeration areas, a total of 9,183 study participants were included. After adjustment with sample weights, the national prevalence of schistosomiasis was 25.6% (95% confidence interval (CI): 22.3, 29.0) with children ages two to four most at risk for the disease with 36.1% infected (95% CI: 30.1, 42.2). The defecation behaviors of an individual were more strongly associated with infection status than the household water and sanitation infrastructure, indicating the importance of incorporating behavior change into community-led total sanitation coverage.

**Conclusions/Significance:**

Our results highlight the importance of incorporating monitoring and evaluation data into control programs in Uganda to understand the geographic distribution of schistosomiasis prevalence outside of communities where endemicity is known to be high. The high prevalence of schistosomiasis among the youngest age group, ineligible to receive drug treatment, shows the imperative to develop a child-appropriate drug protocol that can be safely administered to preschool-aged children. Water and sanitation interventions should be considered an essential investment for elimination alongside drug treatment.

## Author summary

Schistosomiasis is a neglected tropical disease in sub-Saharan Africa that has remained intractable despite efforts to eliminate it through mass drug administration. The transmission cycle is perpetuated when sanitation infrastructure does not adequately capture infected urine or feces and local water bodies, with snail vectors, are contaminated. Schistosomiasis has been linked with stunting and cognitive deficits and there is particular concern for the most vulnerable age group under five years old who are undergoing critical intestinal development but are ineligible to receive drug treatment. Efforts to reduce the disease have focused on children and young adolescents in endemic areas, near water bodies where transmission is known to be high. In Uganda, where fresh water bodies are abundant and intestinal schistosomiasis is endemic, very little is understood about the disease prevalence at a national level. We conducted a large, nationally representative survey and found a national prevalence of 25.6% where the 2–4 year old children had the highest prevalence for schistosomiasis with 36.1% infected. The most significant risk-factor for the disease was an individual’s open defecation behaviors in surface waters. This emphasizes the need to include water and sanitation investments alongside drug treatment and behavior change to control schistosomiasis in Uganda.

## Introduction

Schistosomiasis is a neglected water-based, vector-borne disease transmitted indirectly through freshwater snails, estimated to affect more than 240 million individuals worldwide with 700–800 million people living at risk of infection [1–3]. In sub-Saharan Africa, approximately 280,000 deaths per year have been attributed to schistosome infections and the onset of complications caused by the disease [4]. The transmission cycle of the disease is perpetuated when infected individuals defecate (predominately *Schistosoma mansoni*) or urinate (predominately *Schistosoma haematobium*) in open waters and the miracidia, released from hatched schistosome eggs, infect suitable snail hosts [5]. The cercariae are then subsequently expelled from the snails and infect individuals in contact with cercariae-contaminated waters via penetration through the skin. The lack of sanitation infrastructure to adequately collect and treat human waste, along with the common behaviors and activities that involve frequent contact with open waters, has made schistosomiasis a challenging disease to control in sub-Saharan Africa.

Approaches to estimate the scale of the disease have primarily focused on measuring disease prevalence in areas where endemic status is known to be high, particularly in shoreline communities where water contact is frequent [6–13]. In Uganda, where the disease is considered endemic, the control program has focused mainly on chemotherapeutic intervention using praziquantel with less emphasis on interrupting environmental transmission [14]. In 2018, coverage of either annual or biennial mass drug treatment, coupled with health education, was achieved across all 82 districts where schistosomiasis is common. Despite this progress, there is doubt that the targets set by the World Health Organization will be met to control morbidity by 2020 and achieve elimination by 2025 [15]. To accurately assess progress toward schistosomiasis elimination in Uganda a comprehensive assessment of disease prevalence and intensity across the population is necessary.

To understand the prevalence of schistosomiasis in Uganda and the associated risk factors, we undertook the first nationally representative survey, which included testing with detection of worm antigens (circulating cathodic antigen–CCA) in urine samples, diagnosis and treatment of schistosomiasis-positive participants within sampled households. The aims of this study were to assess the prevalence of schistosomiasis across Uganda and characterize the relationship between infection with *S*. *mansoni* (the predominant circulating strain in Uganda [16–18]) and its associated risk factors to help develop sustainable strategies to control schistosomiasis in Uganda.

## Methods

### Ethics statement

This manuscript has been developed according to the consolidated standards of observational studies (see S1 STROBE checklist). The study received ethical clearance from the Institutional Review Boards at Makerere University School of Public Health (Kampala, Uganda; reference no. 424), Johns Hopkins University (Baltimore, Maryland reference no. IRB00007024), the Uganda National Council for Science and Technology (UNCST; Kampala, Uganda; reference no. HS 2069) and with permission from the Uganda Office of the President and district authorities. The following enrollment procedures were approved by the ethical committees: all participants or caregivers were informed about the purpose and procedures of the study and they were invited to sign a written consent form. In case of illiteracy, participants were asked to record their thumbprint alongside a literate witness. Those with informed consent were assigned a unique identifier. Diagnostic results were communicated to participants and those found positive for infection with schistosomiasis, and who met criteria for treatment (e.g., a single 40 mg/kg oral dose of praziquantel using height measured against a tablet pole as a proxy), were treated according to national guidelines [19].

### Study design

We conducted rapid data collection using mobile technology under the Performance Monitoring & Accountability 2020 (PMA2020) platform that utilizes a multi-stage cluster design with fixed, census-derived enumeration areas (EAs) and random selection of households [20]. To determine an accurate national prevalence rate for schistosomiasis in Uganda a three-tiered, stratified, clustered, random sampling was employed. The first tier of sampling randomly selected EAs according to their classification of either nearby or far from water bodies. The second tier of random sampling selected households within the EAs by first mapping the borders and occupied households within the selected EA. Once all households within the EA boundary were mapped and listed, a random sample of 30 households was made using a random number generator application. The third tier of sampling randomly selected individuals within each sampled household and tested them for schistosomiasis. All household members were listed during the household interview and were either usual members of the household or slept in the household the night before. A random number generator was used to randomly select an individual from the roster as a study participant. Therefore, no adjustment is made for the probability of selecting an individual from within the household. At all tiers, the sampling probabilities were available because the sample frame for all households and eligible individuals within each household was known.

Monitoring of schistosomiasis in Uganda has historically focused on school-based sampling within districts, which results in a large variance and high design effect due to the strong intra-cluster correlation [21]. The sampling strategy for this study incorporated community distances from water bodies, such as wetlands and surface water sources to categorize an EA as near (“endemic”) or far (“non-endemic”). In 2004, Kabatereine *et al*. found *S*. *mansoni* prevalence rates, using egg counts in stool with the Kato-Katz method, were higher in communities that were 5km or less from the shores of Lake Victoria or Lake Albert and lower in communities more than 5 km [22]. In collaboration with the Uganda Bureau of Statistics (UBOS), a nationally representative sample of EAs was randomly selected, which are geographic units of a size determined by UBOS that average 100 households. EAs were sampled based on their proximity to water bodies based on if their boundaries fall within (near) or outside of (far) 5 km of wetlands and water bodies. To determine an EA’s classification, UBOS established the distance to a water body based on detailed nationwide GIS data that included the boundaries of all seasonal wetlands and surface water bodies as small as 10m in one dimension. Rice paddies and similar wetland crops were not included because water intensive crops are expected to be in proximity or co-located with natural wetlands and surface water bodies.

To determine the sample size a conservative assumption was used that the prevalence rate among individuals living in households in the near strata is 50%, leading to the largest possible sample size for that strata. For EA clusters over 5 km from water bodies in the far strata, 15% prevalence was assumed. We used 80% power and 5% type-I error and applied an estimated design effect of four. We adjusted the final sample size to account for non-response to reach a target of 170 EAs. The final proportion of EAs was chosen according to UBOS’s cartography unit estimate of approximately 70% of the Ugandan population near (within 5 km of) a water body for a final number of 120 EAs in the near strata and 50 in the far strata. With 30 households in each EA selected over two consecutive years, we calculated a sample size of 7,200 in the near strata and 3,000 in the far strata for a sample size target of 10,200. To collect the sample a pooled cross-sectional design over the two years of 2016 and 2017 was necessary to measure the effect of praziquantel drug treatment on the subset of study participants with a positive diagnosis for schistosomiasis in the first survey round. The sample was pooled to enable more precise estimates in sub-analyses where sample sizes would be limited if only one survey round was used.

### Setting

Data were collected from 170 EAs between October to December in 2016 and from the same set of EAs in the same season in 2017. Two different random samplings of households were selected for each year to allow for a pooled cross-sectional analysis where data are combined from 2016 and 2017. The surveys were scheduled to precede the mass drug administration (MDA) planned by Ministry of Health Vector Control Division (MOH/VCD) in order to ensure that measurement of the national prevalence rate was not affected by the implementation of community-wide drug treatments. Community leaders in each EA were asked if MDA had ever been previously implemented in their community.

### Household questionnaire

For each household randomly selected for the survey a household questionnaire was administered by a trained enumerator fluent in the local language. The head of the household or a competent household member was interviewed for questions relevant to the household. The household questionnaire collected information on socio-demographic factors related to wealth, education, construction quality of the dwelling, and water, sanitation and hygiene (WASH) characteristics. The PMA2020 platform is designed for collection of WASH indicators [23, 24] and was adapted for collection of these variables in the context of schistosomiasis.

### Individual questionnaire

One household member, two years of age and older, was randomly selected to participate in the study. The individual questionnaire included a series of questions on WASH practices and open defecation behaviors, knowledge of schistosomiasis and uses of nearby water bodies that may expose the individual to schistosomiasis. At the end of the questionnaire the individual was tested for schistosomiasis. If the randomly selected individual in the household was a child under 8 years old then the primary caregiver of the child was interviewed. For children ages 8–17 the caregiver was present to assist the child with questions regarding the child’s knowledge, attitudes and practices with regards to schistosomiasis and the caregiver was interviewed regarding the child’s schistosomiasis infection history and demographics. This ensured that the most appropriate person was interviewed to obtain the most accurate information possible regarding schistosomiasis and the child.

### Urine collection and analysis

After the individual questionnaire was completed, respondents were asked to provide a urine sample in a sterile cup. If the individual was not able to provide a sample at the conclusion of the questionnaire, the enumerator came back at a time when the sample could be provided. Enumerators were trained by personnel from MOH/VCD to administer a rapid, commercially available antigen test, the Point-of-Care Circulating Cathodic Antigen test (POC-CCA, Rapid Medical Diagnostics, Pretoria, South Africa), which measures the CCA of juvenile and adult *S*. *mansoni* that are released into the circulation. The test has been recommended by the WHO for screening of intestinal schistosomiasis and its protocol, with interpretation by color change, was well suited for training the enumerators [25]. The quantity of this antigen in urine correlates with the worm burden to indicate an active infection [26]. The POC-CCA test has been shown to have higher sensitivity than the standard Kato-Katz method when it was evaluated in endemic settings in the Africa region [27–31]. The POC-CCA test was conducted according to the manufacturer’s guidelines and was repeated a second time if the first test was considered invalid. Test results were scored as negative if the CCA band was absent and positive if present. Cases with trace results for CCA were considered as positive to most accurately estimate infection prevalence [32]. The tests were interpreted independently by enumerators and recorded by photo on the mobile devices for validation.

### Diagnosis and treatment

Participants with a positive *S*. *mansoni* diagnosis by POC-CCA were treated for schistosomiasis with oral doses of praziquantel if they met the criteria for treatment as advised by the Uganda Ministry of Health. Children under five years of age found to be positive were given albendazole (400 mg) but were not treated with praziquantel and breastfeeding or pregnant women were also not treated, per MoH/VCD guidance.

### Water body mapping

Water body locations were recorded by field workers at each EA to understand the relationship between prevalence rates using CCA and proximity to water bodies, which may harbor the intermediate snail hosts. To measure individual distances between households and water bodies located within one kilometer of an EA boundary, field workers utilized the global positioning system (GPS) features on their mobile phones and recorded a GPS point for a water body based on the most likely place of entry into the surface water. GPS points were also recorded for the household during the household questionnaire and the difference between these two locations was calculated. If more than one water body was recorded for an EA then the median distance calculated for that household was analyzed as the distance between the household and a water body.

### Data handling and quality assurance

All administered questionnaires had their responses directly entered into an Android smartphone using Open Data Kit (ODK) software similar to protocols from the PMA2020 program [20]. Following the interview, testing, diagnosis and treatment, household and individual questionnaires were submitted to a secure cloud server, where they were aggregated and monitored on a daily basis by data managers and quality assurance teams at Makerere University School of Public Health in Kampala. Technical assistance was provided by the PMA2020 team at Johns Hopkins University. To ensure data quality, the following procedures were put in place: i) the POC-CCA tests were randomly replicated in spot checks by supervisors to validate the diagnosis of infection status; ii) enumerators recorded their start and stop times that could later be cross-checked by the ODK data to verify that the diagnosis from the POC-CCA cassette was within the specified time window; iii) photos of the POC-CCA test were recorded and verified with the diagnosis entered into ODK by quality assurance teams; and iv) a set of questions were selected for monitoring of data quality during data collection by the quality control team.

### Data analysis

The national *S*. *mansoni* prevalence estimate was determined as the number of individuals testing CCA positive for schistosomiasis divided by the total number of participants with test results available. The national estimate was weighted using the adjusted sample weights. Pearson’s χ2 was used to test for differences of prevalence estimates across categories. Population wealth was measured using quintiles that were calculated using a pre-determined set of asset variables collected from the household questionnaire. An open defecation/urination (OD/U) index was constructed from the individual questionnaire where “low” represented no self-reported OD/U, “medium” indicated OD/U in the bush only and “high” indicated OD/U in open water bodies. The independent effect of factors associated with the CCA prevalence of *S*. *mansoni* infection was determined using robust “modified” Poisson regression and the results are reported as unadjusted and adjusted prevalence ratios (PRs) and 95% confidence intervals (CI), along with the test for significance. Given the common occurrence of schistosomiasis prevalence (greater than 10%) prevalence ratios are reported to be “conservative, consistent, and interpretable” [33–35]. To account for clustering at the EA level, unadjusted and adjusted multilevel mixed-effects generalized linear models were applied using a "modified Poisson" approach to estimate the prevalence ratios for schistosomiasis risk-factors [36]. The adjusted model was built by first including risk-factor variables that are well established including sex, age group and the interaction between sex and age group [37–39]. Risk factors were then entered together with sex, age group and the interaction between the two and if significant (using likelihood ratio test) were added to the adjusted model [18]. The final adjusted model fit was assessed using both the deviance statistic and the Pearson statistic generated from the goodness-of-fit test [40]. P-values less than 0.05 were considered significant. Statistical analyses were done using STATA version 14.0 (Stata Corporation; College Station, United States of America). Maps were created in QGIS Geographic Information System 3.0.2 Girona Open Source Geospatial Foundation Project (http://qgis.osgeo.org). EA locations were geolocated by creating a centroid using the GPS coordinate data collected at each household listed in the EA and for confidentiality, the centroid GPS location was randomly displaced.

## Results

### Study participation

Enumerators reached all of the 170 randomly sampled EAs pre-selected for inclusion in the two-year serial cross-sectional survey. Household questionnaires were completed for 9,516 households (completion rate 93.3%) with the majority of non-responses due to not being at home (2.6%, n = 265) and refusal (1.4%, n = 146). Individual questionnaires were completed for 9,183 randomly selected household members (completion rate 96.5%) with the majority of non-responses due to not being at home (2.2%, n = 208) and refusal (0.7%, n = 63). The diagnostic urine test was successfully completed by 99% of the individuals who accepted to be interviewed for a total of 9,097 individuals included in the sample.

### Prevalence of schistosomiasis by socio-demographic characteristics of the population

**Fig 1:**
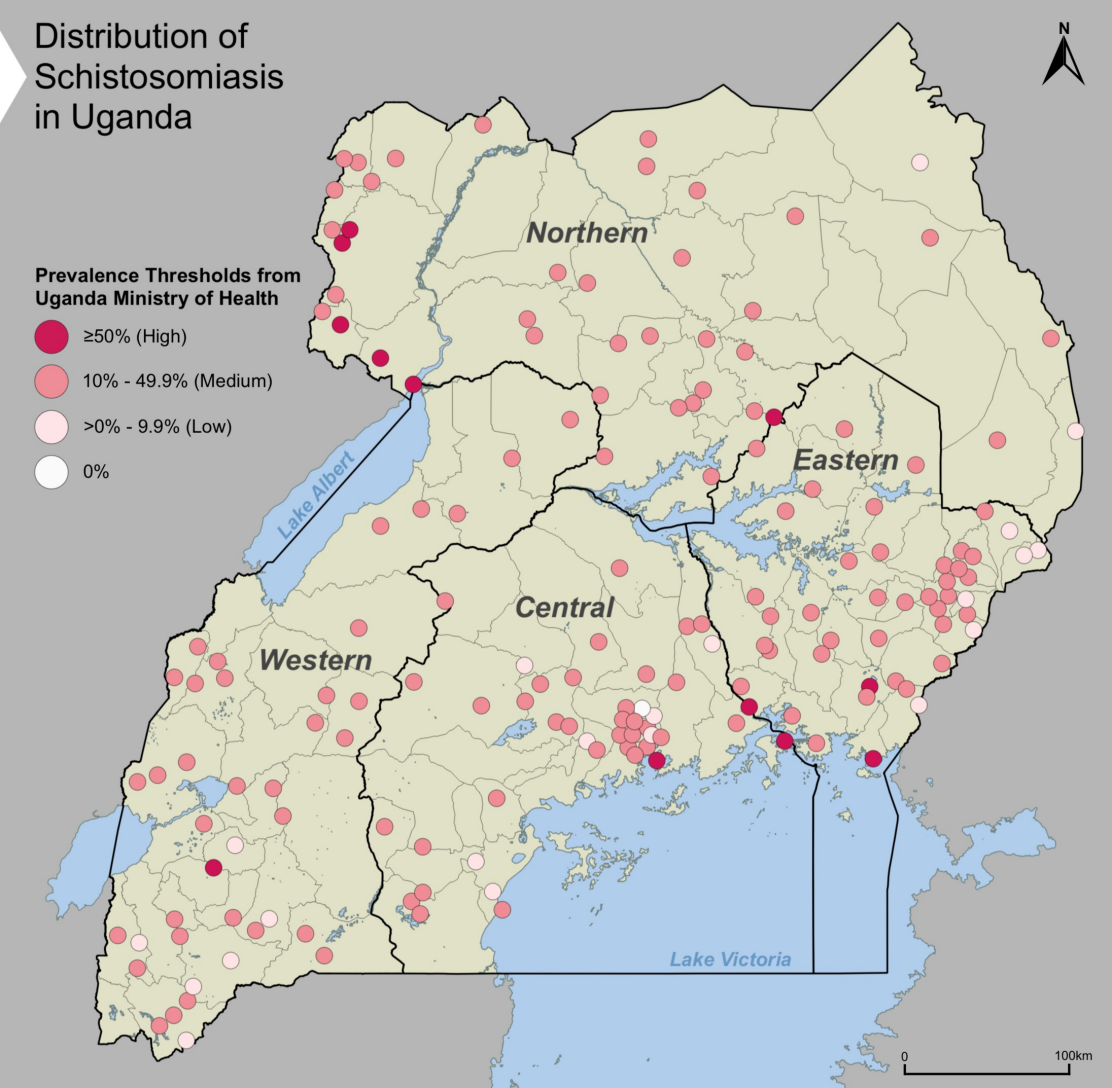
Distribution of schistosomiasis among participants in each of the 170 randomly sampled enumeration areas in Uganda.

The national prevalence of schistosomiasis using CCA was 25.6% (95% confidence interval (CI): 22.3, 29.0) after probability sample weighting. Disaggregated by year the 2016 national prevalence was 22.1% (95% CI: 18.7, 25.6) and 2017 national prevalence was 29.0% (95% CI: 24.4, 33.6). Fig 1 presents the percent of participants who tested positive for schistosomiasis in each of the 170 randomly sampled EAs in Uganda.

Comparisons of disease prevalence using CCA by socio-demographic categories show the greatest differences for age, religion, household size, sex, region and whether the EA received mass drug administration in years prior to the survey (yes/no). Table 1 presents the percent of probability sample weighted estimates for those infected with schistosomiasis by the socio-demographic categories. The age group with the highest prevalence for schistosomiasis was the 2–4 year old children with 36.1% infected (95% CI: 30.1, 42.2). For subsequent age groups, 27.7% of children age 5–10 years old were infected (95% CI: 23.4, 32.0) and 30.9% of 11–15 year olds were infected (95% CI: 25.8, 36.1). The prevalence of schistosomiasis steadily declined with each consecutive older age group. There were no differences in infection status by education levels and marginal differences by wealth quintiles.

**Table 1:** Schistosomiasis prevalence using CCA in Uganda by socio-demographic characteristics; probability sample weighted estimates (N = 9,097).

Characteristics	Prevalence percentage (95% CI)	n	p-value (Pearson’s χ2)
Age (years)			p < 0.0001
2–4	36.1 (30.1, 42.2)	967	
5–10	27.7 (23.4, 32.0)	1520	
11–15	30.9 (25.8, 36.1)	896	
16–20	29.1 (24.6, 33.6)	982	
21–30	25.4 (21.0, 29.7)	1872	
31–40	23.6 (16.9, 30.4)	1019	
41–50	19.7 (13.5, 25.8)	712	
50+	11.9 (7.7, 16.2)	1129	
Sex			p = 0.012
Male	27.5 (23.9, 31.1)	4234	
Female	24.2 (20.6, 27.7)	4863	
Region			p = 0.020
Northern	31.5 (24.3, 38.6)	2162	
Western	19.8 (15.0, 24.5)	2291	
Eastern	30.4 (23.7, 37.0)	2519	
Central	24.6 (17.1, 31.0)	2125	
Wealth quintile			p = 0.051
Lowest	26.4 (20.7, 32.2)	1997	
Lower	27.7 (23.1, 32.3)	1922	
Middle	24.9 (21.2, 28.6)	1738	
Higher	28.7 (23.3, 34.0)	1771	
Highest	20.2 (15.6, 24.9)	1669	
Education (>18 years old) N = 5177			p = 0.097
None	18.1 (13.0, 23.2)	954	
Primary	23.8 (18.9, 28.7)	2759	
Secondary or more	20.4 (16.3, 24.5)	1464	
Household size (# of members)			p = 0.004
1–2	22.0 (18.2, 25.9)	2556	
3–4	26.4 (22.1, 30.8)	2739	
5–6	25.4 (21.8, 29.0)	2159	
>7	30.4 (25.6, 35.1)	1643	
Religion			p = 0.0005
Catholic	23.7 (20.4, 26.9)	3738	
Protestant	22.7 (18.6, 26.9)	2828	
Muslim	34.6 (26.7, 42.4)	1213	
Pentecostal	28.1 (21.1, 35.0)	781	
Seventh Day Adventists	28.0 (16.4, 39.5)	171	
Other	37.2 (29.7, 44.7)	328	
Enumeration area received mass drug administration in years prior			p < 0.0001
No	23.7 (20.4, 27.1)	7165	
Yes	37.8 (32.0, 43.5)	1516	

**Fig 2:**
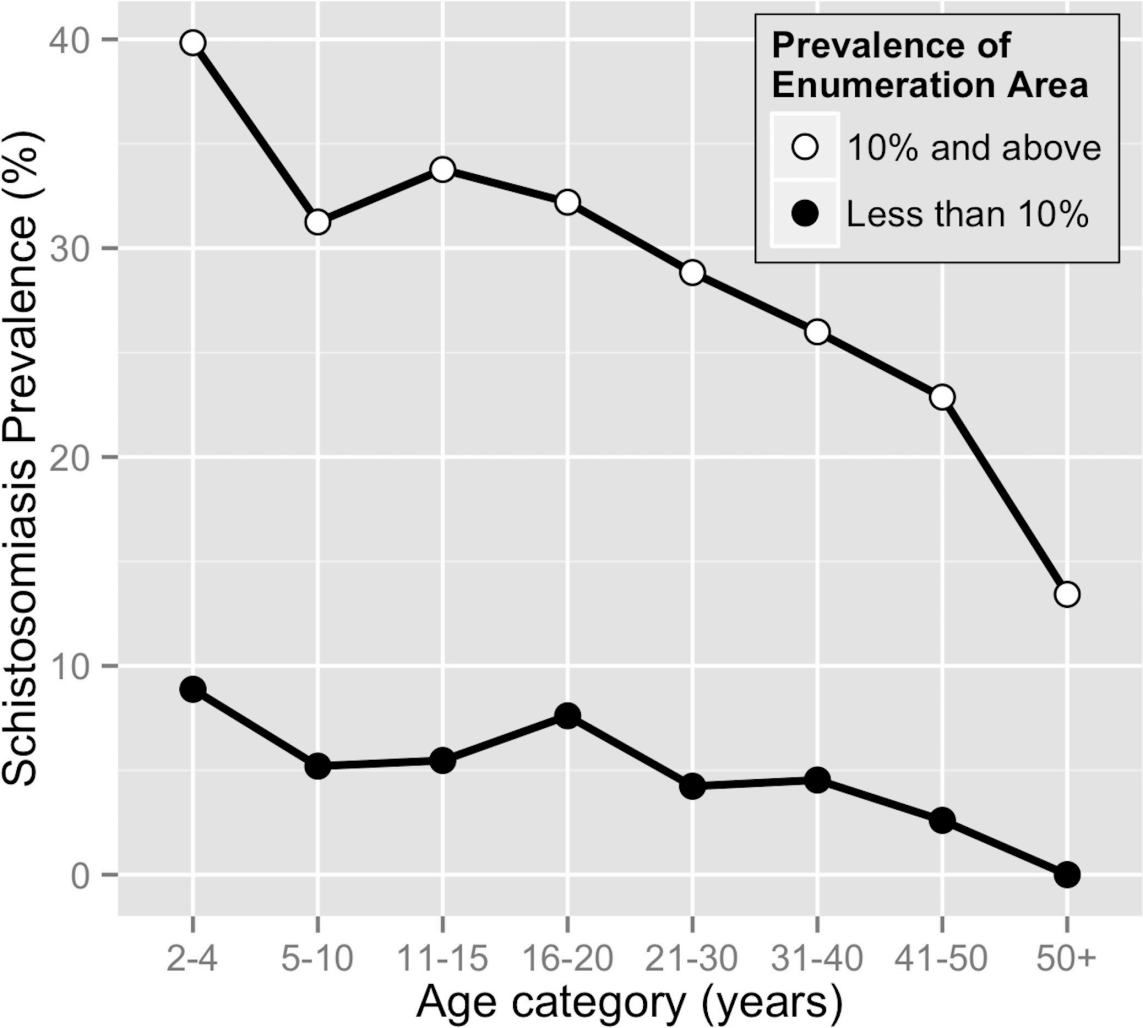
Age prevalence CCA curves for high prevalence enumeration areas (> = 10%, n = 7,857) and low prevalence enumeration areas (<10%, n = 1,157).

Age prevalence CCA curves in Fig 2 show the different burdens of disease by ages for high prevalence EAs, considered to be 10% or greater, and low prevalence EAs, considered to be less than 10%. The age groups were divided into 2–4 years old to represent the preschool aged children ineligible to receive praziquantel drug treatment. Subsequent ages were grouped in five year increments to analyze the impact of school-based drug administration: the 5–10 and 11–15 year olds most likely to receive drug treatment through the school-based drug administrations, and the 16–20 year olds who most likely no longer have access to school-based drug treatment programs. The older age groups, less likely to receive yearly access to mass drug administration, were grouped in ten year increments. The 2–4 years old group for both curves were the most at-risk group for schistosomiasis with 39.8% infected in the high prevalence EAs and 8.9% infected in the low prevalence EAs.

### Prevalence of schistosomiasis by water and sanitation characteristics

Household water and sanitation characteristics were recorded from all 9,097 of the study participants. The prevalence of schistosomiasis using CCA did not significantly vary by household water and sanitation categories (S1 Table) including main drinking water source classified by the Joint Monitoring Programme [41] as “improved” or “unimproved”, use of surface water for any daily purpose (e.g. drinking, washing, cooking, bathing) and classification as near or far from a water body.

The prevalence of schistosomiasis using CCA did vary based on individual water and sanitation practices (S2 Table). Schistosomiasis was significantly higher among those who self-reported to defecate in surface water (31.2% (95% CI: 25.2, 37.2) versus those that did not self-report the practice (24.1% infected (95% CI: 20.8, 27.5) (p = 0.0063)). For individuals self-reporting to submerge in surface water, defined as putting hands, feet, or any other part of the body into a water body, within the last year, 26.6% were infected (95% CI: 22.7, 30.6) versus those that reported not to submerge themselves with 21.5% infected (95% CI: 17.7, 25.4) (p = 0.0133). The prevalence of schistosomiasis significantly increased with each level of the OD/U index: low likelihood of OD/U had 18.4% infected (95% CI: 13.9, 23.0), medium likelihood 24.8% infected (95% CI: 21.6, 28.0) and high likelihood 29.3% infected (95% CI: 24.3, 34.4)) (p = 0.0005 for Chi-square for trend test).

**Fig 3:**
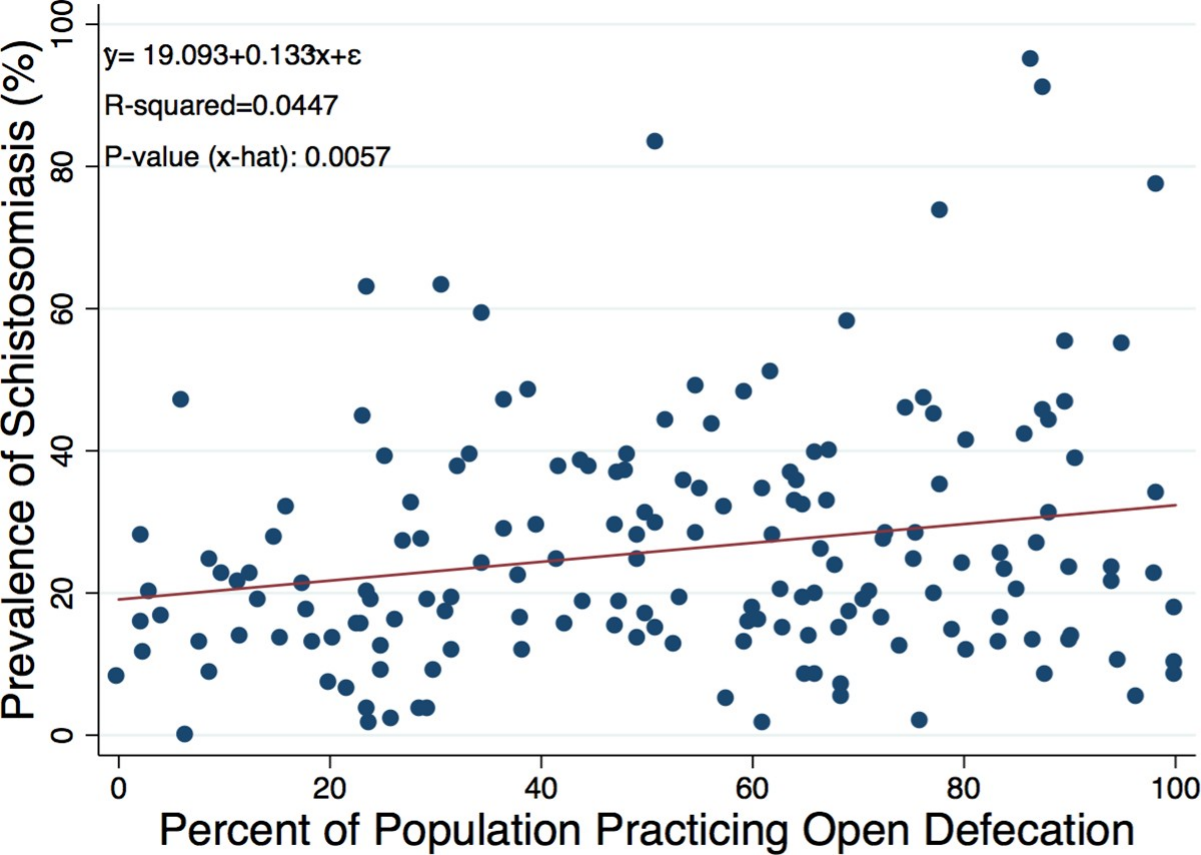
Community open defecation rates (percent of individuals in an enumeration area stating that they openly defecate in the bush or in water bodies) versus the prevalence of schistosomiasis using CCA in an enumeration area (n = 170).

Fig 3 shows the community open defecation rates (percent of individuals in a given enumeration area stating that they openly defecate in the bush or in water bodies) versus the prevalence of schistosomiasis using CCA in that same enumeration area and shows an increasing trend in disease prevalence as the percent of open defecation in the community increases (n = 170).

### Knowledge of schistosomiasis in the population

The percentage of the sampled population that had heard of schistosomiasis prior to the survey was 61.8% (n = 5570) (S3 Table). Those that had previously heard of schistosomiasis had a significantly higher prevalence of schistosomiasis infection using CCA (28.4% (95% CI: 24.3%,32.5%) versus those who had not heard of the disease (21.1% (95% CI: 17.9%, 24.2%; p = 0.0002). The activity that respondents most frequently identified as being a risk factor for schistosomiasis infection was wading into water to wash clothes, fetch water or bathe oneself (n = 2075 respondents); there was no difference in disease prevalence between those who knew this was an activity that risked infection and those who did not know.

### Risk factors associated with schistosomiasis infection

Table 2 shows the prevalence ratios for risk factors of schistosomiasis infection (unadjusted and adjusted) among study participants in Uganda. The variables included in the final adjusted model, in addition to sex, age group, and the interaction between sex and age group, were wealth status, region, OD/U index, distance from the household to a water body, and whether the EA had received mass drug administration prior. The prevalence ratios of infection were significantly lower at all ages when compared to children 2–4 years. For children in the 5–10 year old age group, there was a reduction of approximately 48% in the prevalence of schistosomiasis (0.52 aPR, 95% CI: (0.36, 0.77)) compared to the 2–4 year olds. The household water and sanitation characteristics were not significant risk factors for infection with schistosomiasis while the individual water and sanitation related behaviors were found to be risk factors. For individuals with the highest likelihood to openly defecate and or urinate in a water body (High OD/U index), compared to those with the lowest likelihood (Low OD/U index), they were 1.36 times more likely to be infected with schistosomiasis (1.36 aPR, 95% CI: (1.15, 1.61). For every increase in a kilometer of distance there was a reduction in approximately 5% of schistosomiasis prevalence (aPR = 0.95; 95% CI: (0.92, 0.99)). Residence in an EA that received mass drug administration prior to the survey was associated with higher risk of schistosomiasis infection (aPR 1.19, 95% CI: (1.01, 1.41)).

**Table 2:** Prevalence ratios for risk-factors of schistosomiasis infection. Unadjusted and adjusted multilevel mixed-effects generalized linear models with robust variance estimates using a "modified Poisson" approach to estimate the prevalence ratios for schistosomiasis risk-factors among study participants in Uganda from households with water bodies mapped for their EA; clustering accounted for at the EA level. Adjusted model includes interaction term for age and sex.

Characteristics	PR (95% CI) N = 7,927	aPR (95% CI) N = 7,594
Age (years)		
2–4	1.0	1.0
5–10	0.73 (0.64, 0.84)***	0.52 (0.36, 0.77)**
11–15	0.80 (0.68, 0.93)**	0.64 (0.41, 1.00)*
16–20	0.78 (0.68, 0.90)***	0.86 (0.61, 1.22
21–30	0.75 (0.65, 0.86)***	0.73 (0.52, 1.03
31–40	0.66 (0.56, 0.77)***	0.59 (0.40, 0.88)*
41–50	0.53 (0.43, 0.65)***	0.36 (0.21, 0.56)***
50+	0.29 (0.23, 0.36)***	0.17 (0.11, 0.26)***
Sex		
Female	1.0	1.0
Male	1.11 (1.02, 1.19)*	1.33 (0.91, 1.95)
Household size (# of members)	1.03 (1.02, 1.05)***	—
Region		
Northern	1.0	1.0
Western	0.63 (0.47, 0.84)**	0.64 (0.41, 0.98)*
Eastern	0.82 (0.62, 1.07)	0.77 (0.53, 1.11)
Central	0.68 (0.51, 0.92)*	0.81 (0.52, 1.28)
Wealth quintile		
Lowest	1.0	1.0
Lower	0.94 (0.84, 1.06)	0.96 (0.76, 1.20)
Middle	0.95 (0.84, 1.07)	0.97 (0.74, 1.28)
Higher	0.95 (0.84, 1.07)	1.05 (0.81, 1.37)
Highest	0.85 (0.74, 0.98)*	0.98 (0.72, 1.33)
Religion		
Muslim	1.0	—
Catholic	0.84 (0.75, 0.94)**	—
Protestant	0.89 (0.79, 1.00)	—
Pentecostal	0.98 (0.85, 1.12)	—
Seventh Day Adventist	0.82 (0.59, 1.16)	—
Other	1.06 (0.90, 1.27)	—
Has heard of schistosomiasis		
No	1.0	—
Yes	1.03 (0.95, 1.12)	—
Main drinking water source classification		
Improved	1.0	—
Unimproved	0.99 (0.88, 1.10)	—
Use of surface water for household purposes		
No	1.0	—
Yes	1.08 (0.95, 1.23)	—
Main household sanitation classification		
Improved, not shared facility	1.0	—
Shared facility	1.10 (0.99, 1.87)	—
Non-improved facility	1.10 (0.94, 1.53)	—
Open defecation	1.09 (0.92, 1.29)	—
Self-reported open defecation/urination index		
Low	1.0	1.0
Medium	1.23 (1.05, 1.43)**	1.18 (0.99, 1.39)
High	1.43 (1.21, 1.68)***	1.36 (1.15, 1.61)***
Individual self-reports submerging oneself in surface water within the last year		
No	1.0	—
Yes	1.18 (1.04, 1.33)**	—
Median distance from household to water body		
1 km increase in distance	0.95 (0.92, 0.98)**	0.95 (0.92, 0.99)**
Enumeration area received mass drug administration		
No	1.0	1.0
Yes	1.18 (0.95, 1.45)	1.19 (1.01, 1.41)*
Education (>18 years old)^1^		
None	1.0	—
Primary	1.40 (1.20, 1.63)***	—
Secondary or more	1.37 (1.14, 1.64)**	—

*p-value < 0.05;

**p-value < 0.01;

***p-value < 0.001

^1^ Education not include in adjusted model due to age cut-off over 18 years old

### Water body mapping and associations with schistosomiasis

A total of 433 water bodies were identified from field work that were recorded to be within 10 kilometers of a household. There was a decreasing trend in the prevalence of schistosomiasis as the household distance from a water body increased (S1 Fig). The decreasing trend is also at the community level where prevalence of schistosomiasis decreases by 1.1% for every increase in a kilometer of mean distance between water bodies and the households in an EA (S2 Fig).

## Discussion

This study found a national prevalence of schistosomiasis across Uganda at 25.6% (95% CI: 22.3, 29.0) with CCA testing by using a large, representative sample of EAs and probability sample weights. The study had complete adherence to the random sample reaching all 170 pre-selected EAs by UBOS and found that intestinal schistosomiasis is prevalent and widespread across Uganda, even outside of areas known to be endemic. This is in line with a similar finding from western Tanzania where distribution of *S*. *mansoni* was more widespread than was previously understood [42]. This was the first national survey in Uganda to investigate schistosomiasis beyond known endemic areas and adds to work on the epidemiology of *S*. *mansoni* in Uganda by Kabatereine *et al*. that purposively selected communities in areas near large water bodies, where prevalence was expected to be high [22]. The known areas of high prevalence were included at random into the study sample and resulted in a national prevalence with CCA testing that averaged these areas of high transmission with areas of lower transmission. The finding that the Northern and Eastern regions of Uganda had higher prevalence is consistent with previous work that focused on the more endemic areas [12, 22, 43]. Prior estimates suggested that in Uganda 4 million individuals were infected with schistosomiasis caused by *S*. *mansoni* [44] and this study increases that estimate to over 10 million. This has implications for treatment programs to increase coverage across Uganda. The higher prevalence in the communities that had received mass drug administration prior to the survey (37.8% (95% CI: 32.0%, 43.5%)) indicates that the communities already receiving treatment may need more intense and focused efforts to bring the disease under control.

Children of preschool age, from 2 to 4 years old, were identified as the most at-risk age group with a schistosomiasis prevalence of 36.1% (95% CI: (30.1, 42.2), n = 980) using CCA testing. This improves our understanding of the disease in this age group, which is often ignored in schistosomiasis control work given their ineligibility to receive drug treatment. The high prevalence found in preschool aged children supports prior studies that identified this age group as particularly important to treat to reduce heavy infections and early cumulative morbidity as they progress through childhood [45, 46]. Early treatment of this at-risk age group could also mitigate the educational, learning, and memory deficits that *Schistosoma* infection and non-treatment have been found to be associated with in school aged children [47]. The high prevalence rate among children aged 2 to 4 years old also provides insight into how national control campaigns, that focus on drug delivery to school aged children, may have shifted the peak of age-based infection profiles to the preschool age groups, which previously peaked among school-aged children [1, 22, 48]. Preschool aged children may be spreading schistosomiasis as they engage in high risk activities for contaminating water bodies such as bathing and playing in surface water while their caregivers wash clothes [49]. The practical implication of this is that school-based treatments may be insufficient to reduce the disease burden in children [50] and there is a need to formulate a child-appropriate praziquantel tablet that can be safely administered to preschool aged children in control programs [51]. This high disease burden highlights the need for equitable treatment of young children to close the “praziquantel treatment gap” and reduce *Schistosoma* transmission in the general population [52]. As demonstrated in this study, preschool aged children should be treated with a praziquantel formulation in areas found to be highly endemic with CCA-based surveys [53].

The primary risk factors for *S*. *mansoni* that had strong associations with infection were an individual’s open defecation behaviors. Improved water and sanitation infrastructure in a household was not associated with reduced infection, indicating that the presence of adequate sanitation does not necessarily guarantee its use [54]. This is particularly important in the context of schistosomiasis where the bulk of the eggs reaching the freshwater snails are thought to stem from direct urination or defecation, largely by children that are bathing and swimming in the open water [55]. To sustain schistosomiasis transmission only a few eggs are required to enter freshwater and therefore a small number of individuals can continue the transmission cycle. For *S*. *mansoni* eggs that can survive out of water for up to eight days [56], feces left in the bush or in latrines near water bodies may be washed in from rains or trodden into water bodies by people or animals [57]. The finding from this study that lower rates of open defecation in a community are associated with lower schistosomiasis disease prevalence is consistent with previous work that found increased latrine coverage along with a decrease in community wide open defecation reduced schistosomiasis prevalence [58]. In Uganda past surveying and mapping efforts have identified hotspots for schistosomiasis and other soil-transmitted helminths that pose a particular risk in the context of low sanitation coverage [59].

The protective relationship found between a household’s increasing distance from a water body and an individual’s infection with schistosomiasis confirms that human surface water use and surface water contact behaviors are contributing factors of disease transmission [60]. Notably, this protective relationship was found for individuals living in households up to 10 kilometer distances from water bodies, which is more geographically widespread than national control programs typically administer mass drug treatment. There was no significant difference in disease prevalence using CCA testing found between the near and far strata used for sampling EAs. The implication for this is that future sampling strategies should focus more on continuous distances from water bodies rather than dichotomous categories. Much of lakeside Uganda is characterized by largely mobile, itinerant fishing communities, whose frequent movements spread intestinal schistosomiasis outside of these hotspot areas and may also result in their children missing school-based annual mass drug administration [61]. The freshwater habitats for the intermediate snail hosts have also been found outside of targeted areas for control where natural transmission is thought unlikely [62, 63]. For Uganda this requires control program implementation on a much larger scale than has been previously executed given that the majority of communities in Uganda are accessible to freshwater.

A strength of the study is that the sampling frame allows the results to be interpreted as a national prevalence estimate for the Ugandan population. This was made possible by the sampling design and probability weights used from the collaboration with UBOS. Similar national prevalence surveys using CCA testing could be undertaken in other countries in the African Great Lakes Region plagued with high endemicity of schistosomiasis. Bureaus of statistics should be consulted to use a multi-stage cluster design to randomly sample EAs based on distances from water bodies and provide probability sample weights. Another strength was the use of POC-CCA, a commercialized urine-based antigen test that is used specifically for the detection of active *S*. *mansoni* infection in humans [64]. The use of POC-CCA enabled the large, nationwide scale of the study with its ease of use under field conditions and minimal training requirements for its application [65]. The use of urine samples instead of stool also increased its acceptability by respondents which make it ideal for epidemiological surveys in large populations [66]. The POC-CCA test has been suggested to be more sensitive than the Kato-Katz stool smears, which have historically been used for disease diagnosis in previous studies [67, 68]. Related to this, the main limitation of the study is that the POC-CCA detection method may over-estimate prevalence in low prevalence areas due to cross-reactions with other helminth infections to produce false-positives [68]. The inclusion of lower intensity infections may have also over-estimated prevalence in low endemic areas where individuals without active schistosomiasis show residual reactivity (trace) POC-CCA [69]. This is also relevant in the context of control programs where the intensity of infection as measured by Kato-Katz is an important measure and one that is not available using POC-CCA. In a recent study that compared diagnostic methods to detect *S*. *mansoni*, the POC-CCA cassette test was recommended for moderate and high prevalence areas and used for screening and geographical mapping of *S*. *mansoni* infections [70]. This has also been validated in school-aged children in endemic settings around Lake Victoria to show that it is an appropriate and effective means of rapidly testing for intestinal schistosomiasis [29].

The current targets for eliminating schistosomiasis in Uganda by 2020 are most likely not going to be met as indicated by the national prevalence rate of 25.6% found in this study and stated by others in the literature [71]. There is a clear imperative for more accurate data to determine the treatment strategies for all age groups and incorporate the scale up of water and sanitation interventions [60, 72, 73]. To accelerate progress toward elimination of schistosomiasis in Uganda there is a need to strengthen evidence on how to deliver effective WASH interventions for neglected tropical diseases and break down existing programming silos built around single interventions [74, 75].

## Supporting information

S1 Checklist STROBE statement—Checklist of items that should be included in reports of cross-sectional studies. (DOC)

S1 File Raw data file. (CSV)

S1 Table Schistosomiasis prevalence in Uganda by household water and sanitation-related characteristics (probability sample weighted estimates). (DOCX)

S2 Table Schistosomiasis prevalence in Uganda by individual water and sanitation-related characteristics (probability sample weighted estimates). (DOCX)

S3 Table Comparisons between schistosomiasis knowledge and prevalence of disease (probability sample weighted estimates). (DOCX)

S1 Fig Prevalence of schistosomiasis in relationship to the median distance a household was located from a water body in their enumeration area. (DOCX)

S2 Fig Mean distance between households in an enumeration area and a water body versus the prevalence of schistosomiasis in an enumeration area (n = 159). (DOCX)
