# Supplementary material for: The prevalence of schistosomiasis in Uganda: A nationally representative population estimate to inform control programs and water and sanitation interventions
Source: PLoS Negl Trop Dis. 2019 Aug 14;13(8):e0007617. doi: 10.1371/journal.pntd.0007617 (PMC6709927; doi:10.1371/journal.pntd.0007617)
Supplement: S1 Checklist — (DOC) [file pntd.0007617.s001.doc]

STROBE Statement—Checklist of items that should be included in reports of ***cross-sectional studies***

|  | Item No | Recommendation |
| --- | --- | --- |
| **Title and abstract** | 1 | (*a*) Indicate the study’s design with a commonly used term in the title or the abstract  The title “The prevalence of schistosomiasis in Uganda: A nationally representative population estimate to inform control programs and water and sanitation interventions” uses common terms and indicates that national level surveillance of schistosomiasis was done in the study. |
|  |  | (*b*) Provide in the abstract an informative and balanced summary of what was done and what was found  Refer to lines 29-46 of the manuscript. It provides a summary of the key methods used and main findings. |
| Introduction | | |
| Background/rationale | 2 | Explain the scientific background and rationale for the investigation being reported  The introduction to the manuscript starts with a general description of schistosomiasis and narrows down to the relevant information that justifies the current study (lines 81-107). |
| Objectives | 3 | State specific objectives, including any prespecified hypotheses  The objective of the study was to assess the prevalence of schistosomiasis across Uganda and characterize the relationship between infection with *S. mansoni* and its associated risk factors (lines 115-118). No prespecified hypotheses are declared. |
| Methods | | |
| Study design | 4 | Present key elements of study design early in the paper  The key elements of study design have been presented on lines 142-172 |
| Setting | 5 | Describe the setting, locations, and relevant dates, including periods of recruitment, exposure, follow-up, and data collection  Refer to lines 175-181 of the manuscript for the information on the setting, locations relevant dates for recruitment and data collection |
| Participants | 6 | (*a*) Give the eligibility criteria, and the sources and methods of selection of participants  The study was designed to randomly sample the population across all socio-demographic profiles and ages (above 2 years old). A three-stage random sampling strategy was utilized to select the subjects who participated in the study (lines 186-201) |
| Variables | 7 | Clearly define all outcomes, exposures, predictors, potential confounders, and effect modifiers. Give diagnostic criteria, if applicable  In this present survey, prevalence was the outcome variable while exposure and confounding variables are described on lines 188-199. Diagnostics used in the study are described in full on lines 204-226. |
| Data sources/ measurement | 8* | For each variable of interest, give sources of data and details of methods of assessment (measurement). Describe comparability of assessment methods if there is more than one group  All data from this survey were obtained from questionnaire based primary data collection (lines 186-201). |
| Bias | 9 | Describe any efforts to address potential sources of bias  To rule out bias, random sampling was employed to select the subjects who participated in the study. |
| Study size | 10 | Explain how the study size was arrived at  The sample size calculation is described on lines 159-168. |
| Quantitative variables | 11 | Explain how quantitative variables were handled in the analyses. If applicable, describe which groupings were chosen and why  The quantitative variables were expressed as % (prevalence) and the variables used in the questionnaires are described on lines 186-201 |
| Statistical methods | 12 | (*a*) Describe all statistical methods, including those used to control for confounding  All statistical methods used in the survey are clearly stated in lines 266-287. |
|  |  | (*b*) Describe any methods used to examine subgroups and interactions  Subgroup and interactions were examined that had been previously established in the literature as cited in lines 276-278. |
|  |  | (*c*) Explain how missing data were addressed  Not applicable |
|  |  | (*d*) If applicable, describe analytical methods taking account of sampling strategy  Not applicable |
|  |  | (*e*) Describe any sensitivity analyses  Not applicable |
| Results | | |
| Participants | 13* | (a) Report numbers of individuals at each stage of study—eg numbers potentially eligible, examined for eligibility, confirmed eligible, included in the study, completing follow-up, and analysed  A complete discussion of study participation is on lines 292-305 |
|  |  | (b) Give reasons for non-participation at each stage  See lines 292-305 |
|  |  | (c) Consider use of a flow diagram  This was deemed not necessary for inclusion in the manuscript |
| Descriptive data | 14* | (a) Give characteristics of study participants (eg demographic, clinical, social) and information on exposures and potential confounders  See Table 1 for study participant characteristics and descriptions on lines 318-330 |
|  |  | (b) Indicate number of participants with missing data for each variable of interest  Not applicable |
| Outcome data | 15* | Report numbers of outcome events or summary measures  Refer to Tables 1-4 |
| Main results | 16 | (*a*) Give unadjusted estimates and, if applicable, confounder-adjusted estimates and their precision (eg, 95% confidence interval). Make clear which confounders were adjusted for and why they were included  Refer to Table 5 for adjusted and unadjusted prevalence ratio estimates |
|  |  | (*b*) Report category boundaries when continuous variables were categorized  Age categorizations can be found in Table 1 |
|  |  | (*c*) If relevant, consider translating estimates of relative risk into absolute risk for a meaningful time period  Not applicable |
| Other analyses | 17 | Report other analyses done—eg analyses of subgroups and interactions, and sensitivity analyses  These have been addressed above |
| Discussion | | |
| Key results | 18 | Summarise key results with reference to study objectives  Key results were summarised in lines 447-449, 465-467, and 484-485 |
| Limitations | 19 | Discuss limitations of the study, taking into account sources of potential bias or imprecision. Discuss both direction and magnitude of any potential bias  Limitations are discussed on lines 525-528. |
| Interpretation | 20 | Give a cautious overall interpretation of results considering objectives, limitations, multiplicity of analyses, results from similar studies, and other relevant evidence  Interpretations of results from this survey are directly addressed in lines 533-541. |
| Generalisability | 21 | Discuss the generalisability (external validity) of the study results  The study is valid/applicable to Uganda and therefore not generalizable beyond that. |
| Other information | | |
| Funding | 22 | Give the source of funding and the role of the funders for the present study and, if applicable, for the original study on which the present article is based  This work was funded by Maxmind, Inc in partnership with Performance Monitoring and Accountability 2020 and the Bill and Melinda Gates Institute for Population and Reproductive Health at the Johns Hopkins School of Public Health. The funders had no involvement in the concept, design, conduct, analysis or interpretation of this study. |

*Give information separately for exposed and unexposed groups.

**Note:** An Explanation and Elaboration article discusses each checklist item and gives methodological background and published examples of transparent reporting. The STROBE checklist is best used in conjunction with this article (freely available on the Web sites of PLoS Medicine at http://www.plosmedicine.org/, Annals of Internal Medicine at http://www.annals.org/, and Epidemiology at http://www.epidem.com/). Information on the STROBE Initiative is available at www.strobe-statement.org.
