# Supplementary material for: The prevalence of schistosomiasis in Uganda: A nationally representative population estimate to inform control programs and water and sanitation interventions
Source: PLoS Negl Trop Dis. 2019 Aug 14;13(8):e0007617. doi: 10.1371/journal.pntd.0007617 (PMC6709927; doi:10.1371/journal.pntd.0007617)
Supplement: S1 Table — (DOCX) [file pntd.0007617.s003.docx]

Table 2. Schistosomiasis prevalence in Uganda by household water and sanitation-related characteristics; probability sample weighted estimates (N=9,097).

| Household water and sanitation-related characteristics | Prevalence percentage  (95% CI) | n | p-value (Pearson’s χ2) |
| --- | --- | --- | --- |
| Main drinking water source classification |  |  | p = 0.909 |
| Improved | 25.5 (21.9, 29.1) | 2363 |  |
| Unimproved | 25.9 (20.0, 31.8) | 6727 |  |
| Use of surface water for household purposes |  |  | p = 0.582 |
| Yes | 27.6 (18.5, 36.7) | 1652 |  |
| No | 25.1 (22.0, 28.3) | 7445 |  |
| Main household sanitation classification |  |  | p = 0.527 |
| Improved, not shared facility | 21.2 (15.3, 27.2) | 952 |  |
| Shared facility | 27.6 (20.9, 34.3) | 1131 |  |
| Non-improved facility | 26.0 (22.4, 29.6) | 6124 |  |
| Open defecation | 25.1 (16.7, 33.4) | 882 |  |
| Household is within 5km of a water body^1^ |  |  | p = 0.568 |
| Yes | 26.2 (22.2, 30.1) | 7548 |  |
| No | 23.6 (16.2, 31.0) | 1549 |  |

^1^ Distance measured by enumerators during data collection
