# Supplementary material for: The prevalence of schistosomiasis in Uganda: A nationally representative population estimate to inform control programs and water and sanitation interventions
Source: PLoS Negl Trop Dis. 2019 Aug 14;13(8):e0007617. doi: 10.1371/journal.pntd.0007617 (PMC6709927; doi:10.1371/journal.pntd.0007617)
Supplement: S2 Table — (DOCX) [file pntd.0007617.s004.docx]

Table 3. Schistosomiasis prevalence in Uganda by individual water and sanitation-related characteristics; probability sample weighted estimates (N= 8,966).

| Individual water and sanitation-related characteristics | Prevalence percentage  (95% CI) | n | p-value (Pearson’s χ2) |
| --- | --- | --- | --- |
| Individual self-reports defecating in surface water |  |  | p = 0.0063 |
| Yes | 31.2 (25.2, 37.2) | 2080 |  |
| No | 24.1 (20.8, 27.5) | 6984 |  |
| Individual self-reports submerging oneself in surface water within the last year |  |  | p = 0.0133 |
| Yes | 26.6 (22.7, 30.6) | 6294 |  |
| No | 21.5 (17.7, 25.4) | 1887 |  |
| Open defecation/urination index |  |  | p = 0.0005^a^ |
| Low | 18.4 (13.9, 23.0) | 1205 |  |
| Medium | 24.8 (21.6, 28.0) | 4215 |  |
| High | 29.3 (24.3, 34.4) | 3628 |  |

^a^ Chi square test for trend p-value <0001
