# Supplementary material for: The prevalence of schistosomiasis in Uganda: A nationally representative population estimate to inform control programs and water and sanitation interventions
Source: PLoS Negl Trop Dis. 2019 Aug 14;13(8):e0007617. doi: 10.1371/journal.pntd.0007617 (PMC6709927; doi:10.1371/journal.pntd.0007617)
Supplement: S3 Table — (DOCX) [file pntd.0007617.s005.docx]

Table 4. Comparisons between schistosomiasis knowledge and prevalence of disease (probability sample weighted estimates).

| Knowledge about schistosomiasis | Prevalence percentage  (95% CI) | n | p-value (Pearson’s χ2) |
| --- | --- | --- | --- |
| Has heard of schistosomiasis |  | 9006 | p=0.0002 |
| Yes | 28.4 (24.3, 32.5) | 5570 |  |
| No | 21.1 (17.9, 24.2) | 3436 |  |
| Self-reported ever having schistosomiasis^^[[1]](#footnote-1)^^ |  | 4825 | p=0.228 |
| Yes | 33.0 (22.7, 43.3) | 287 |  |
| No | 27.5 (23.4, 31.6) | 4538 |  |
| Knowledge of activities that risk infection with schistosomiasis^1^ |  | 5043 |  |
| Wading to wash clothes, fetching water or bathing |  |  | p=0.931 |
| Yes | 28.1 (22.7, 33.4) | 2075 |  |
| No | 28.3 (23.6, 33.0) | 2968 |  |
| Swimming |  |  | p=0.052 |
| Yes | 18.6 (9.8, 27.5) | 171 |  |
| No | 28.5 (24.2, 32.9) | 4872 |  |
| Working in water (e.g. boating, farming, fishing) |  |  | p=0.045 |
| Yes | 33.9 (26.4, 41.4) | 383 |  |
| No | 27.7 (23.5, 32.0) | 4660 |  |
| Drinking contaminated water^^[[2]](#footnote-2)^^ |  |  | p= 0.317 |
| Yes | 30.5 (24.9, 36.1) | 1359 |  |
| No | 27.4 (22.5, 32.2) | 3684 |  |
| Don’t know^^[[3]](#footnote-3)^^ |  |  | p=0.357 |
| Yes | 26.9 (21.7, 32.2) | 1730 |  |
| No | 28.9 (24.5, 33.3) | 3313 |  |
| Knowledge of how schistosomiasis is transmitted from one person to someone else^^[[4]](#footnote-4)^^ |  | 5043 |  |
| Infected person urinating near water |  |  | p=0.217 |
| Yes | 25.4 (20.0, 30.7) | 846 |  |
| No | 28.8 (24.2, 33.3) | 4197 |  |
| Infected person defecating near water |  |  | p=0.374 |
| Yes | 25.6 (19.0, 32.2) | 568 |  |
| No | 28.5 (24.1, 33.0) | 4475 |  |
| Don't know^^[[5]](#footnote-5)^^ |  |  | p=0.207 |
| Yes | 29.4 (24.2, 34.7) | 3030 |  |
| No | 26.4 (22.1, 30.6) | 2013 |  |

1. Only asked of those who had heard of the disease [↑](#footnote-ref-1)
2. This is an incorrect answer given the low likelihood of cercarie infecting an individual through lips while drinking water. [↑](#footnote-ref-2)
3. If the participant did not know the answer to the question this was recorded as “Yes”. The “No” category indicates at least one of the other categories was selected. [↑](#footnote-ref-3)
4. Only asked of those who had heard of the disease [↑](#footnote-ref-4)
5. If the participant did not know the answer to the question this was recorded as “Yes”. The “No” category indicates at least one of the other categories was selected. [↑](#footnote-ref-5)
