# Supplementary material for: The prevalence of schistosomiasis in Uganda: A nationally representative population estimate to inform control programs and water and sanitation interventions
Source: PLoS Negl Trop Dis. 2019 Aug 14;13(8):e0007617. doi: 10.1371/journal.pntd.0007617 (PMC6709927; doi:10.1371/journal.pntd.0007617)

S1 Figure. Prevalence of Schistosomiasis in relationship to the median distance a household was located from a water body in their enumeration area. These data in the blue line reflect the modeled data in Table 2 with the 95% confidence intervals in gray. The cut-off of 10km was used for data analysis because distances beyond this point were considered too far to interact with a water body on a basis necessary for transmission and water bodies were not mapped by enumerators at these large distances.


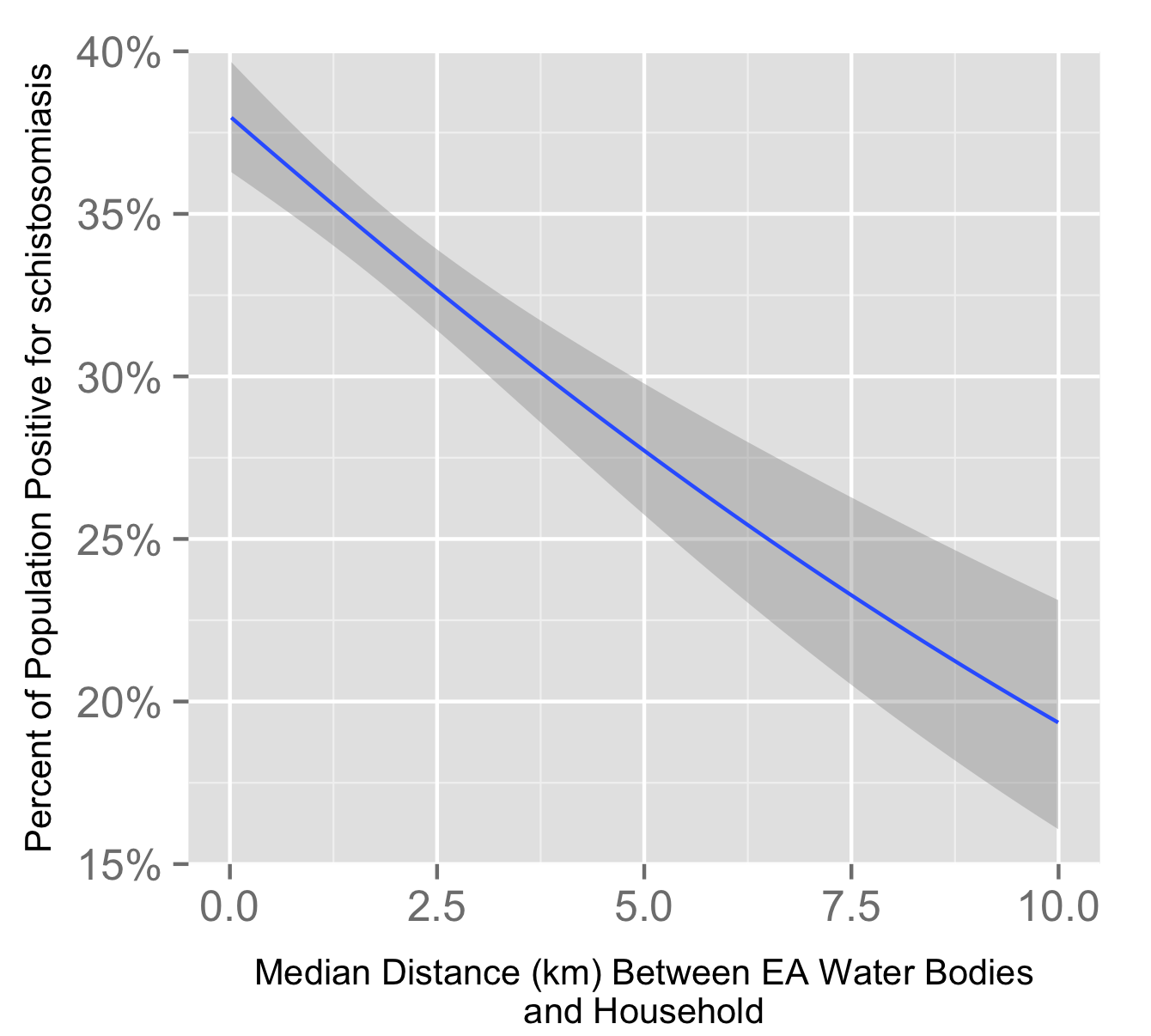

Supplement: S1 Fig — (DOCX) [file pntd.0007617.s006.docx]
