# Supplementary material for: The prevalence of schistosomiasis in Uganda: A nationally representative population estimate to inform control programs and water and sanitation interventions
Source: PLoS Negl Trop Dis. 2019 Aug 14;13(8):e0007617. doi: 10.1371/journal.pntd.0007617 (PMC6709927; doi:10.1371/journal.pntd.0007617)

S2 Figure. Mean distance between households in an enumeration area and a water body versus the prevalence of schistosomiasis in an enumeration area (n=159). Sample size was reduced due to lack of presence of a water body within 1 km of the boundary of an enumeration area.


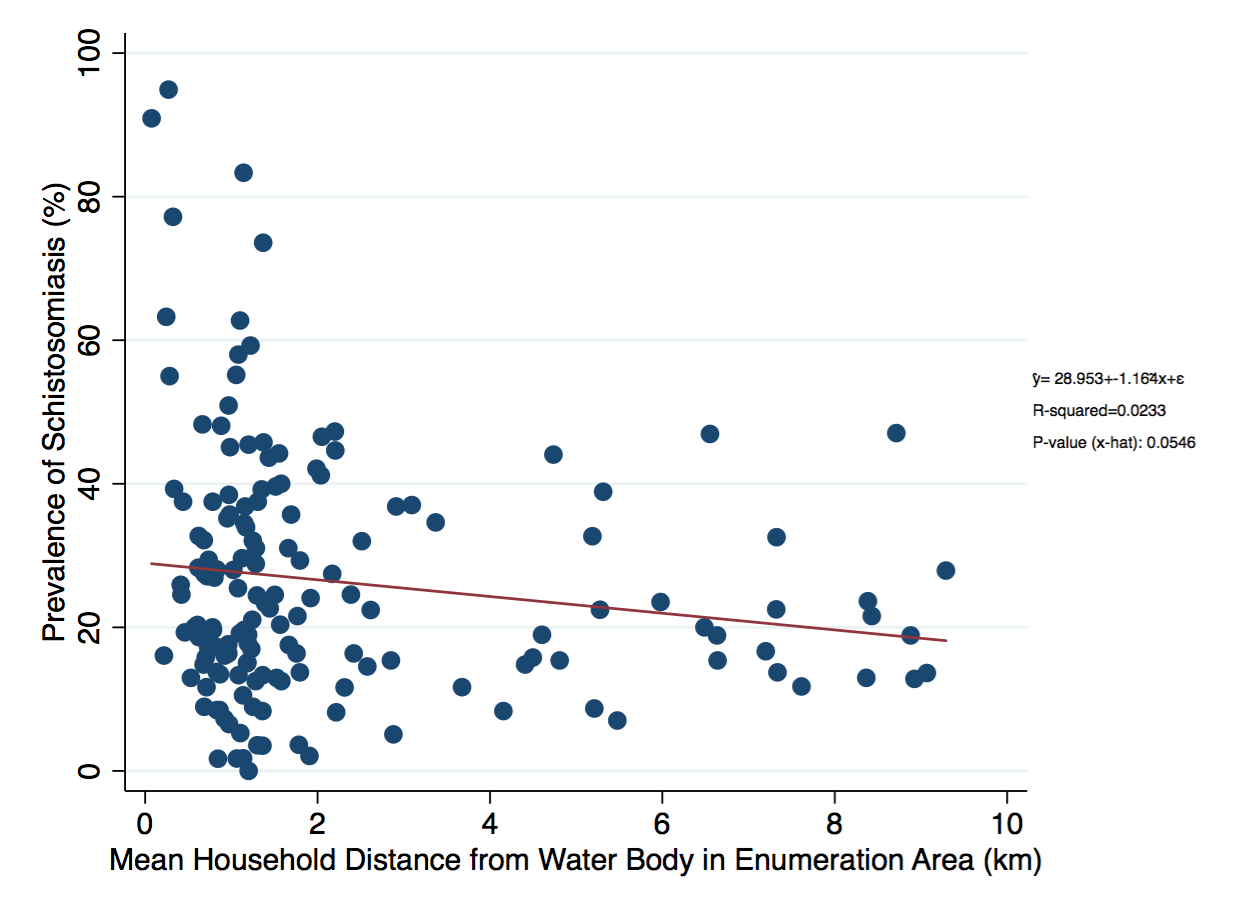

Supplement: S2 Fig — (DOCX) [file pntd.0007617.s007.docx]
